# Dislocation of Total Hip Replacement in Femoral Neck Fracture: Do Surgical Approach and Dual Mobility Implant Matter?

**DOI:** 10.7759/cureus.21031

**Published:** 2022-01-08

**Authors:** Poornanand Goru, Syed Haque, Gopalkrishna G Verma, Abubakar Mustafa, Mostafa Hamed, Mobeen Ismail, Sanat Shah

**Affiliations:** 1 Trauma and Orthopaedics, Manchester University NHS Foundation Trust, Manchester, GBR

**Keywords:** dislocation, dual mobility, single bearing, dislocation prosthesis, total hip replacement (thr), fracture neck of femur

## Abstract

Introduction: Total hip replacement (THR) in the neck of femur fracture in the elderly is associated with a higher risk of dislocation compared to hemiarthroplasty of hip or total hip replacement in the native hip. There is uncertainty regarding combining surgical approach, femoral head size, and the usage of single bearing or dual mobility to reduce the risk of dislocation. This study looks into the bearing of the prosthesis for posterior or lateral surgical hip approach as well as their head size to give a stable hip to these vulnerable groups of patients.

Methods: Initial data were collected retrospectively from February 2017 till May 2019 from the electronic records database and clinical notes. Patients included in the study had a femoral neck fracture (age >60 years) who underwent a total hip replacement. Subsequent data were collected prospectively from June 2019 to July 2020.

Results: High rate of dislocation was found with posterior approach and single bearing prosthesis. However, if dual mobility prosthesis was used while using the posterior approach the dislocation rate was very low. Also, with lateral approach and single bearing prosthesis using large femoral head size, the dislocation rate was negligible.

Conclusions: We recommend a dual mobility prosthesis for posterior approach THR and lateral approach with single-bearing hip replacement with large size femoral head. The dislocation rate is low using this principle irrespective of the surgical approach.

## Introduction

Approximately 1.6 million hip fractures occur worldwide each year and by 2050 this number could reach between 4.5 million and 6.3 million [1]. Around 76,000 patients are admitted with fractured neck of femur (NOF) in the United Kingdom every year, nearly half of these sustain a displaced intracapsular fracture NOF [2]. The National Institute for Health and Care Excellence (NICE) has produced guidance that suggests replacement arthroplasty with either hemiarthroplasty or total hip replacement (THR) in patients with displaced intracapsular NOF [3]. It further recommends offering THR in patients who were able to walk independently out of doors with no more than the use of a stick and are not cognitively impaired and are medically fit for anesthesia and the procedure.

However, in 2018 only a third of patients were eligible as per NICE guidelines and had THR [3]. There are many reasons for widespread non-compliance with the NICE guidelines. Total hip replacement carries higher risks than hemiarthroplasty, such as greater intraoperative blood loss, longer operative times, and more postoperative complications. Studies of hemiarthroplasty compared with THR for displaced intracapsular hip fractures have shown a significantly higher risk of postoperative hip dislocation after THR [4,5]. The incidence of dislocation in primary THR ranges from 0.12% to 16%, with an overall pooled incidence of 2% to 10% [6]. This number increases significantly up to 22% for THR done for NOF [7] with most series giving a figure of around 5% [8]. The risk of dislocation may be a major reason that deters surgeons from doing THR in patients eligible for THR with NOF [9].

A posterior approach to the hip joint has been traditionally associated with a higher risk of dislocation compared with a lateral approach to the hip in THR [10]. Studies in the past have shown the posterior approach to be the only factor associated with a significantly increased risk of dislocation in NOF patients having THR [4,9]. In fact, for hemiarthroplasty of the hip for NOF, NICE recommends a lateral approach to the hip to reduce the risk of dislocation [3]. However, there are many favorable functional outcomes especially regarding Trendelenburg gait, heterotrophic calcification, and stem mispositioning in patients in whom THR is done using the posterior approach [11,12]. Also, the posterior approach to the hip for THR in patients with NOF is associated with a lower risk of both patient mortality and intraoperative complications compared with the lateral approach [13]. One of the ways of reducing the dislocation in THR is to use a dual mobility (DM) implant [14,15]. The DM design consists of a small femoral head captive and mobile within a polyethylene liner.

In the DM implant, there are two bearing surfaces, and mobilization of the second articulation of the polyethylene allows for DM cups’ extra arc of movement before impingement of 30.5° in flexion, 15.4° in the abduction, and 22.4° in external rotation [16,17]. The DM bearings have been proven to be cost-effective compared with single bearings in patients undergoing revision THR as the risk of dislocation is reduced significantly by the use of dual mobility prostheses [18]. This is particularly true with patients who are less than 70 years old. As the vast majority of THR is done in patients who have a good life expectancy, this prosthesis has the potential of being beneficial for patients with NOF. This study looks into the dislocation rate in THR in NOF patients comparing single bearing and dual mobility regarding posterior and lateral approaches.

## Materials and methods

This is a multi-surgeon study and all five surgeons were fellowship-trained with over 7 years of experience in lower limb arthroplasty. This study was done at a level 1 trauma center in a busy city center hospital. This hospital admits more than 250 NOF patients through its doors every year. More than half of the patients who have intracapsular NOF and are eligible for THR get THR done which is way above the national average [19]. Data were collected from the electronic records database and clinical notes. Patients were included as per our inclusion and exclusion criteria (Table 1).

**Table 1:** Inclusion and exclusion criteria NOF: Neck of femur fracture, THR: Total hip replacement

Inclusion Criteria	Exclusion Criteria
Age >60 years	Age <60 years
NOF treated by THR	Intracapsular NOF treated by internal fixation
Dislocation during hospital admission for the index procedure	Use of any other approach other than a posterior and lateral approach to hip

The study comprised two legs of observation. In the initial or first leg, patients were included from February 2017 to May 2019 who presented with intracapsular NOF and were treated with THR. At this point, after having an internal review it was found that patients who were having THR using the posterior approach with single bearing prosthesis were having a higher rate of dislocation compared with patients having THR using DM. Also, it was found that large femoral head size with lateral approach has a low dislocation rate in the single bearing prosthesis. Hence it was decided to use DM when using the posterior approach and large femoral head when using the anterolateral approach. In the second leg of the study, data were collected prospectively. As the data for this part of the study was drawn from electronic records and didn't necessitate the need to contact patients, it didn't require IRB approval. Patients admitted between June 2019 to July 2020 who presented with intracapsular fracture NOF and had THR done were included in the study.

## Results

Observations from the first leg of the study

Fifty-three patients who underwent THR for NOF fracture were included. Out of these, 30 THR were done using the posterior approach to the hip, and 23 were done using the lateral approach to the hip. Out of these, 43 used single-bearing THR, and 10 used DM prosthesis (Table 2).

**Table 2:** Relationship between surgical approach and dislocation MDM: Modular dual mobility

Total	Surgical Approach	Number of Patients	Dislocation	
53	Posterior	30	6	Single bearing 5 out of 20
				MDM 1 out of 10
	Lateral	23	1	Single bearing 1 out of 23
				MDM 0 out of 0

Surgical Approach and Dislocation

In the posterior approach group of 30 patients who had THR, six had dislocation while they were still in the hospital. In the lateral approach group out of the 23 patients who had single bearing THR only one patient had a dislocation as noted above in Table 2.

Femoral Head Size and Dislocation in the Single Bearing Group

In all the patients who had dislocation following single bearing THR, the femoral head size was 32 (Table 3).

**Table 3:** Relationship between head size surgical approach and dislocation MDM: Modular dual mobility

Prosthesis	Femoral Head Size	Number of Patients	Surgical Approach	Dislocation
Single bearing 43	28	10	Posterior 3	0
			Lateral 5	0
	32	25	Posterior 14	5
			Lateral 11	1
	36	08	Posterior 1	0
			Lateral 7	0
MDM 10		10	Posterior 10	1 *
			Lateral 0	0

Type of Prosthesis and Dislocation

Of the 43 patients in whom fixed-bearing implants were used for THR, six patients had a dislocation. The only dislocation which happened in the DM group was an intra-prosthetic dislocation performed by the posterior approach, which was picked up on a CT scan and needed an open reduction (Table 3).

Observations from the second part of the study done prospectively

A total of 22 cases were included in the study. Out of these 14 were DM hips and eight were single-bearing hip replacements. There were no dislocations in either group of patients (see Table 4).

**Table 4:** Surgical approach and dislocation following implementation of changes (*prosthesis not available on the day - logistic issue) MDM: Modular dual mobility

Surgical Approach	Prosthesis	Total Number	Femoral Head Size		Dislocation
Posterior	MDM	14	N/A		NONE
	Single Bearing	02*	32		
Lateral	MDM	0	N/A		
	Single Bearing	06	36	5	
			28	1	

## Discussion

The ageing of the UK population will give rise to a doubling in the number of osteoporotic fractures including NOF over the next 50 years [20]. About half of NOFs are intracapsular fractures and as per NICE Clinical Guidelines (CG124), a significant proportion of these fractures would need to be treated with THR. Currently, only a third of the patients eligible for THR after NOF are having the desired surgery [3]. One of the reasons quoted is a higher risk of dislocation in patients who have THR compared to hemiarthroplasty of the hip [8]. The hospital cost of dislocation can be quite significant in primary hip replacement in elective settings [21].

However, in THR for NOF, dislocation can be disastrous for surgeons and catastrophic for patients potentially giving rise to a persistently unstable hip and repeated surgery [10]. Reoperations after operative treatment of hip fracture patients are associated with higher costs and inferior survival. Reoperations increased the overall immediate costs of index fractures by nearly 20% [22]. The DM prostheses because of their increased range of movement arc have a lower rate of revision for dislocation compared with single bearing small femoral head THR [23]. Another way to increase the stability of the hip is by increasing the size of the femoral head. The risk of dislocation is lower in larger-bearing femoral heads. Biomechanically large-femoral head single bearing THR has a larger jump distance before dislocation [24]. But the use of large femoral heads seems to be a trade-off between increased stability and decreased total hip arthroplasty (THA) survivorship [25].

Larger heads are also associated with reducing the available thickness of the polyethylene insert in certain cases giving rise to “minimalist inserts” [14]. In the first leg of this study, there was a significantly high rate of dislocation in THR when a single bearing prosthesis was used using a posterior approach to the hip. However, the dislocation rate was practically negligible in patients with THR for NOF who had it using posterior surgical approach and DM implants. The only dislocation with the DM implant was intra prosthetic dislocation which is usually found in the older generation of implants [25]. In the second leg of the study, all patients who were having THR following NOF using posterior approach had DM implant barring a couple of exceptions when the prosthesis was not available. None of the patients had a dislocation. Thus, our study supports previous studies where short-term revision rate was found to be high in THR done using posterior approach compared to other approaches for fracture neck of the femur [23] and goes a step further in suggesting that the risk of dislocation can be reduced by using DM prosthesis.

Another significant inference from this study was that a combination of a lateral approach to the hip with the large femoral head size is a very safe option as far as dislocation is concerned. This study is unique as it compares dislocation rate taking into account femoral head size, surgical approach, and type of prosthesis used whereas most of the previous studies have just taken two of these three variables into account.

Limitations

However, there are certain limitations of this study. In the first part of the study, the data were collected retrospectively. Also, the sample size is small, but we hope that findings from this study would stimulate a larger, possibly prospective multi-centre study.

## Conclusions

This study found that there is a high rate of dislocation when conventional THR is done using the posterior approach. However, this can be reduced significantly by using DM implants. Dual mobility cups should be taken into account when treating a displaced intracapsular fracture of the proximal femur in elderly patients as soon as the indication for a THR has been retained because of its low dislocation rate. Coincidentally, our study also found that lateral approach in conjunction with the single bearing large femoral head also has a very low dislocation rate in this category of patients. Further randomized control studies are required to assess the potential benefit of using dual mobility cups in NOF fracture patients along with future cost analysis.
